# Current state of ethics literature synthesis: a systematic review of reviews

**DOI:** 10.1186/s12916-016-0688-1

**Published:** 2016-10-03

**Authors:** Marcel Mertz, Hannes Kahrass, Daniel Strech

**Affiliations:** 1 Institute of History, Ethics and Philosophy of Medicine, Hannover Medical School, Hannover, Germany; 2 Research Unit Ethics, Institute of History and Ethics of Medicine, University Hospital Cologne, Cologne, Germany

**Keywords:** Systematic review, Literature review, Normative literature, Argument-based literature, Empirical ethics, Bioethics, Literature search, Evidence-based medicine

## Abstract

**Background:**

Modern standards for evidence-based decision making in clinical care and public health still rely solely on eminence-based input when it comes to normative ethical considerations. Manuals for clinical guideline development or health technology assessment (HTA) do not explain how to search, analyze, and synthesize relevant normative information in a systematic and transparent manner. In the scientific literature, however, systematic or semi-systematic reviews of ethics literature already exist, and scholarly debate on their opportunities and limitations has recently bloomed.

**Methods:**

A systematic review was performed of all existing systematic or semi-systematic reviews for normative ethics literature on medical topics. The study further assessed how these reviews report on their methods for search, selection, analysis, and synthesis of ethics literature.

**Results:**

We identified 84 reviews published between 1997 and 2015 in 65 different journals and demonstrated an increasing publication rate for this type of review. While most reviews reported on different aspects of search and selection methods, reporting was much less explicit for aspects of analysis and synthesis methods: 31 % did not fulfill any criteria related to the reporting of analysis methods; for example, only 25 % of the reviews reported the ethical approach needed to analyze and synthesize normative information.

**Conclusions:**

While reviews of ethics literature are increasingly published, their reporting quality for analysis and synthesis of normative information should be improved. Guiding questions are: What was the applied ethical approach and technical procedure for identifying and extracting the relevant normative information units? What method and procedure was employed for synthesizing normative information? Experts and stakeholders from bioethics, HTA, guideline development, health care professionals, and patient organizations should work together to further develop this area of evidence-based health care.

**Electronic supplementary material:**

The online version of this article (doi:10.1186/s12916-016-0688-1) contains supplementary material, which is available to authorized users.

## Background

Decision making in clinical care, public health, biomedical research, and other fields is strongly based on “external” knowledge (e.g., knowledge from clinical trials, health services research, or economic studies). Non-systematic retrieval and appraisal of external information, however, risks several types of bias and therefore diminishes the quality and accountability of decisions. Systematic reviews (SRs) aim to identify and process information from published material in a systematic, transparent, and reproducible manner. Their ultimate goals are to guarantee comprehensiveness and to reduce systematic errors (bias) in the identification and processing of relevant information, and they are therefore conducive to good evidence-based decision making.

Decision making in medicine, research, and health policy often explicitly or implicitly includes normative ethical considerations. For example, should trial participants be granted access to trial drugs after the end of the study? When health professionals and parents disagree about the appropriate course of medical treatment for a child, under what circumstances is the health professional ethically justified in overriding the parents’ wishes? What are ethical arguments for and against sham interventions? Is it allowable to store biological samples and DNA of minors for non-therapeutic research? When is public health surveillance ethical?

Since the rise of scholarly conduct in “applied” ethical analysis in the 1960s and the establishment of institutes for medical ethics, corresponding peer-reviewed journals, conferences, etc., it seems to be unquestioned that normative ethical input in medical and health policy decision making is a professional enterprise that can be more or less appropriate, of high or low quality, etc. However, it is also known that scholars can come to contrasting but equally well-argued conclusions on what is normatively right or wrong, or more or less appropriate [1–3].

Against this background it is surprising that modern standards for evidence-based decision making in clinical care and public health still rely on eminence-based input alone regarding normative ethical information, even though review methodology has been increasingly used in various disciplines and fields.

Scientific communities such as the Cochrane Collaboration, the Campbell Collaboration, and institutions such as the Institute of Medicine (IOM) or the National Institute for Health and Care Excellence (NICE) provide detailed guidance for review methodologies in different fields [4–6]. While these guidelines cover qualitative as well as quantitative research, they do not explicitly mention whether or how current methodological standards apply to normative ethical literature (“normative literature” for short). Similarly, manuals for evidence-based guideline development do not explain how to include ethical issues in a systematic and transparent manner [7]. Recent methodological debate demonstrated the need of knowledge synthesis methods that are specified for particular types of information [8]. But here again, normative ethical information was not acknowledged explicitly.

The ethics literature includes empirical and normative studies on morally challenging topics. Normative literature aims to evaluate or prescribe policies, (moral) reasons, and decisions for or against particular (moral) judgements and policies. Most often, this type of literature can also be described as “argument-based” or “reason-based” literature [9, 10]. The “source material” of ethics research includes (ethical) theory, intuitions, common sense, and scientifically produced empirical data.

Despite the neglect of reviews on normative literature by manuals for the development of clinical guidelines and health technology assessment (HTA), and despite any explicit guidance on methodological particularities, such reviews of normative literature already exist, and scholarly debate on their opportunities and limitations has recently bloomed [10–13].

This study aimed to identify trends in the quantity of published systematic and semi-systematic reviews of normative ethical or “mixed” (empirical and normative ethical) literature, the academic affiliations of corresponding authors, and other review characteristics. The study further particularly assessed how these reviews report on their methods for (1) search, (2) selection, (3) analysis, and (4) synthesis of ethics literature.

## Methods

### Search

**Fig. 1:**
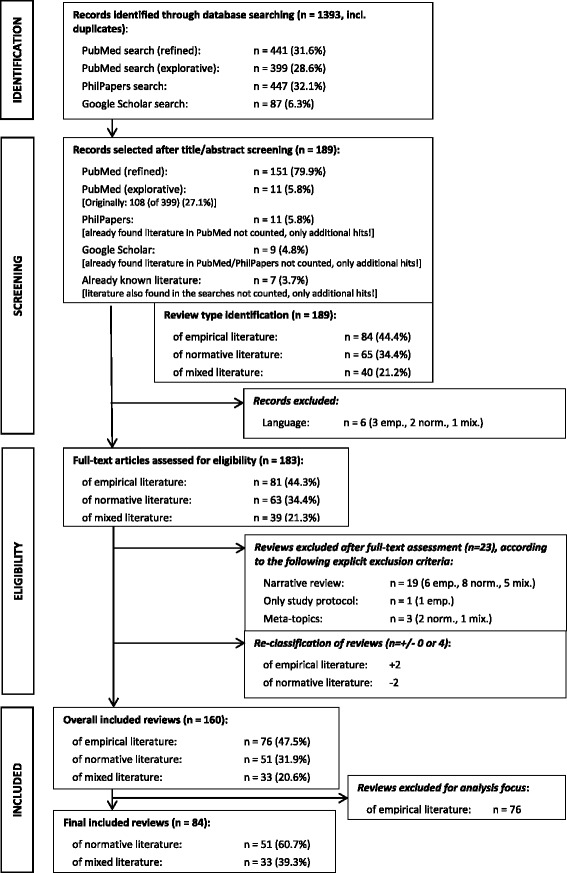
Preferred reporting items for systematic reviews and meta-analyses (PRISMA) flowchart

The review was based on two *PubMed* searches (15 April 2015, 27 April 2015), with additional searches in *PhilPapers* (29 April 2015) and *Google Scholar* (30 April 2015). For *PubMed*, two search strings were used. The first one was composed for screening purposes, and the second one used a refined search string. See Table 1 and the flowchart in Fig. 1.

**Table 1:** Searches and hits

*PubMed* explorative search		
Date:	15 April 2015	
Publication dates:	No restriction (resulted in results from 1988 to 15.04.2015)	
Language:	No restriction	
Search string:	(“systematic review” OR “systematic literature review”[Title/Abstract] OR “qualitative review”[Title/Abstract] OR “literature review”[Title/Abstract] OR “argument-based”[Title/Abstract] OR “systematic survey“[Title/Abstract] OR “literature survey”[Title/Abstract] OR “systematische Übersichtsarbeit“) AND (Ethics[Title/Abstract] OR Bioethics[Title/Abstract] OR “ethical issues”[Title/Abstract] OR normative[Title] OR “ethical guidelines”[Title]) NOT protocol	
Hits:	399	
*PubMed* refined search		
Date:	27 April 2015	
Publication dates:	Start: no date restriction; end: 15.04.2015	
Language:	No restriction	
Search string:	(“systematic review”[Title/Abstract] OR “systematic literature review”[Title/Abstract] OR “qualitative review”[Title/Abstract] OR “literature review”[Title/Abstract] OR “argument-based”[Title/Abstract] OR “systematic survey”[Title/Abstract] OR “systematic search”[Title/Abstract] OR “literature survey”[Title/Abstract] OR “systematische Übersichtsarbeit”) AND ((((Allocat*[Title] OR euthanasia[Title] OR “assisted dying”[Title] OR “end-of-life”[Title] OR palliative[Title] OR ration*[Title] OR attitude*[Title] OR motivation*[Title] OR decision*[Title]) AND (Ethics[Title/Abstract] OR Bioethics[Title/Abstract] OR “ethical issues”[Title/Abstract] OR ethical*[Title/Abstract] OR normative[Title/Abstract] OR “ethical guidelines”[Title/Abstract])) OR (Ethics[Title/Abstract] OR Bioethics[Title/Abstract] OR “ethical issues”[Title/Abstract] OR ethical*[Title] OR normative[Title] OR “ethical guidelines”[Title]))) NOT protocol* NOT “position statement” NOT “ethical approval” NOT ethics commit*	
Explanation:	The exclusion (NOT) conditions for “protocol*”, “position statement”, “ethical approval” and “ethics commit*” were integrated to increase specificity of hits without decreasing sensitivity too much.	
Hits:	441 (incl. duplicate hits regarding earlier search)	
*PhilPapers*		
Date:	29 April 2015	
Publication dates:	Start: no date restriction; end: 15.04.2015 (i.e., all hits after that date ignored)	
Language:	No restriction	
Search mode:	Basic fuzzy filter	
Key words:	Mandatory key words: Relevance key words:	“systematic” AND “review” “ethics”
Hits:	447 (incl. duplicate hits regarding earlier searches)	
*Google Scholar*		
Date:	30 April 2015	
Publication dates:	Start: no date restriction; end: 15.04.2015 (i.e., all hits after that date ignored)	
Language:	No restriction	
Search mode:	Allintitle	
Display mode:	Sorted by relevance; citations deactivated	
Search string:	bioethics OR ethics AND (“systematic review” OR “literature review”)	
Hits:	87 (incl. duplicate hits regarding earlier searches)

It proved to be impossible to search directly and solely for reviews of normative literature, as such a distinction is not established or standardized yet in databases (e.g., no standardized key words refer to this kind of review). Therefore, the search had to be intentionally broad in order to capture any review done related to topics of medical ethics or bioethics, even if this included reviews that solely analyzed and synthesized empirical literature.

We have not used a language restriction for the search in order to assess the overall amount of identifiable reviews.

### Selection

For the purpose of this meta-review on a still little-standardized review area we decided to apply rather sensitive and not too restrictive selection criteria. We selected all reviews that explicitly or implicitly indicated their objective to analyze and present ethics literature in a systematic manner. To be included, reviews had to be *explicitly* concerned with normative ethical considerations of medical topics; e.g., they had to pose an ethical question or determine ethical challenges. It was not deemed sufficient for the results of a review to be able to be regarded as “ethically relevant.” Furthermore, reviews should have an identifiable description of at least some methodological elements describing a reproducible literature search (e.g., search terms, databases used, or inclusion/exclusion criteria). See Table 2. We labeled such reviews as *semi-systematic reviews*. Only those reviews that explicitly or implicitly reported on search, selection, analysis, and synthesis were labeled as *(full) systematic reviews*. Finally, we only included reviews written in English, German, or French.

**Table 2:** Inclusion/exclusion criteria: title/abstract level and full text level

Publication type:	Published journal articles
Language:	Title/abstract level: only articles with at least an abstract in English, German, or French Full text level: only articles in English, German, or French
Content:	The article must:
Inclusion	(a) Be explicitly concerned with normative ethical considerations of medical topics. This meant the article had to, e.g.: i. Pose an ethical question ii. Determine ethical problems/challenges iii. Address ethical decision making or the use of ethical frameworks for decision making iv. Explore ethical views or reasons for/against a decision, etc. v. Look for/produce empirical data for ethical decision making or ethical evaluation vi. Examine ethical regulations or recommendations, etc. It was not sufficient for the abstract to mention, e.g., that the results of the study indicate that there are ethical issues
	(b) Have an identifiable description of at least some methodological elements describing a reproducible literature search (e.g., search terms, databases used, or inclusion/exclusion criteria), irrespective of its own possible labeling as “narrative” or “systematic” review. Only mentioning that “a review was done” was not enough
	(c) Only on full text level: be a review of normative literature or a review of mixed literature.
Exclusion	(a) Not be a review of study protocols or of ethics consultation documentation (b) Not be solely concerned with legal analysis (c) Not solely address “meta”topics of (systematic) reviews, e.g., methodology of literature reviews in bioethics or for ethical aspects in HTA [17, 19–21], methodologies of empirical ethics research [11] or discussions about (research) ethics in (medical) systematic reviews [22] (d) Only on full text level: not be (solely) a review of empirical literature
Quality:	No quality appraisal criteria used (all reviews included that meet the criteria above)

Articles were selected first according to their title or abstract, and later by full text screening. See Table 2. All reviews for empirical, normative, and “mixed” literature were included at this stage. The in-depth analysis and corresponding data presented in this paper focused on the normative and mixed literature, because methodological particularities, especially concerning analysis and synthesis, have been much less widely discussed for normative and conceptual literature than for empirical research.

The selection was initially done by one researcher (MM). Then, a second researcher (HK) checked all the selection results (inclusion and exclusion) for consistency with the selection criteria. Discrepancies were discussed and successfully overcome via consensus-seeking discussions.

Because we aimed to assess the current state of the art of reviews of normative ethical literature, we did not exclude reviews that did not fulfill all PRISMA criteria. Depicting the state of art must also include reviews of “relatively bad” reporting quality. Also, it is possible that certain reviews demonstrate a fair reporting of analysis and synthesis of normative information but are not able to fulfill some basic PRISMA criteria. Excluding such reviews would deprive our review of important insights about how reviews of normative information are analyzing and synthesizing information. Nevertheless, we present slightly adapted PRISMA ratings as part of our results.

Apart from the reporting quality, it would also be impossible to assess the methodological quality of the included reviews because of the lack of specific quality assessment tools for reviews of normative ethics literature.

### Analysis

We determined the academic fields of the journals that published included reviews based on how they were classified by the *Journal Citation Reports* (*JCR*) *Science Edition 2014* and *JCR Social Science Edition 2014*. Where no entry was available, the journal was categorized as “not found”.

We further categorized the affiliation of all authors. (Table 4 lists the different categories used.) For this purpose, we considered the affiliation of all first authors. We took the lowest identifiable organizational unit if several organizational units/levels were mentioned. If the last author had a differing affiliation, this affiliation was also considered. Finally, if additional authors of a review had further differing affiliations, these were also considered. Therefore, the amount of authors considered regarding affiliations is not equal to the total amount of authors.

The method of qualitative content analysis (QCA) [14, 15] was employed to analyze the literature in detail, i.e., to identify and categorize the methods used for search, selection, analysis, and synthesis, and the information given about methodology (e.g., stating aims, discussing limitations, providing a flowchart). In applying this method, we used a combined deductive and inductive strategy for building up categories [14]. This was done iteratively by two researchers (MM, HK).

### Synthesis

The qualitatively analyzed content of the reviews was synthesized into descriptive statistics assessing how often the description of methods corresponded to established (and slightly adapted) criteria of the PRISMA guideline [16] (See Table 6).

## Results

From the initially identified 1393 references we finally included 160 reviews covering three types of ethics reviews: (1) empirical ethics (*n* = 76), (2) normative ethics (*n* = 51), and (3) mixed literature (*n* = 33). For the above-described reasons we further excluded the 76 reviews of empirical ethics literature from the in-depth analysis. See the flowchart in Fig. 1. The following results therefore represent the remaining 84 reviews of normative or mixed literature. Additional file 1: Tables S1–S3 present all references for the three types of ethics reviews.

### Languages, publication dates, and self-labeling

**Fig. 2:**
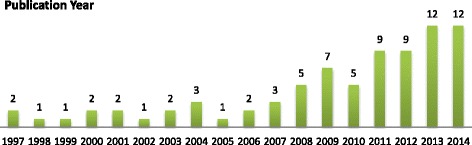
Publication dates of the reviews

Of all 84 reviews, 98 % (*n* = 82) were in English, one in French, and one in German. The earliest reviews were published in 1997. Of the 84 reviews, 82 % were published in the last ten years. See Fig. 2. In total, 31 (37 %) labeled themselves as “systematic review” or used the term “systematic” in labelings such as “systematic literature review” or “systematic survey.”

### Journals: academic fields and titles

The academic fields most prominent were Nursing (*n* = 17, 15 %), Medical Ethics and Ethics (*n* = 10 + 2 = 12, 11 %), Public, Environmental, and Occupational Health (*n* = 8, 7 %), and Genetics and Heredity (*n* = 8, 7 %). See Table 3. Note that a journal can be classified in two or more fields.

**Table 3:** Journals (fields and titles) of the reviews (sorted after highest ranking)

Academic field (according to JCR)	Number of journals (multiple response possible) (*n* = 111)
Nursing	17 (15 %)
Medical Ethics	10 (9 %)
Genetics and Heredity	8 (7 %)
Public, Environmental, and Occupational Health	8 (7 %)
Health Care Sciences and Services	7 (6 %)
Psychiatry	6 (5 %)
Biochemistry and Molecular Biology	4 (4 %)
Medicine, General and Internal	3 (3 %)
Pharmacology and Pharmacy	3 (3 %)
Surgery	3 (3 %)
Ethics	2 (2 %)
Geriatrics and Gerontology	2 (2 %)
History and Philosophy of Science	2 (2 %)
Multidisciplinary Sciences	2 (2 %)
Neurosciences	2 (2 %)
Pediatrics	2 (2 %)
Fields that only came up once*	22 (19 %)
Not found in *JCR Science/Social Science Edition 2014*	8 (7 %)
Journal title	Number of reviews (*n* = 84)
*Nursing Ethics*	7 (8 %)
*BMC Medical Ethics*	4 (5 %)
*European Journal of Human Genetics*	4 (5 %)
*Journal of Advanced Nursing*	4 (5 %)
*Journal of Medical Ethics*	4 (5 %)
*Scandinavian Journal of Caring Sciences*	2 (2 %)
Journals with only one published article	59 (70 %)

*Anesthesiology, Audiology and Speech-Language Pathology, Cell and Tissue Engineering, Cell Biology, Dermatology, Endocrinology and Metabolism, Engineering, Environmental Sciences, Health Policy and Services, Medical Informatics, Medicine, Research and Experimental, Multidisciplinary, Nutrition and Dietetics, Obstetrics and Gynecology, Otorhinolaryngology, Psychology, Rehabilitation, Rheumatology, Social Sciences/Biomedical, Sport Sciences, Substance Abuse, Transplantation

The journal that published the most reviews was *Nursing Ethics* (*n* = 7, 8 %), followed by *Journal of Medical Ethics* (*n* = 4, 5 %), *BMC Medical Ethics* (*n* = 4, 5 %), *Journal of Advanced Nursing* (*n* = 4, 5 %), and *European Journal of Human Genetics* (*n* = 4, 5 %). However, roughly 70 % (*n* = 59) of all finally included reviews (*n* = 84) were found in journals that only appeared once in our review. See Table 3.

### Authors: number, country of origin, and affiliations

The greatest number of reviews were authored by two authors (*n* = 26, 31 %), followed by three (*n* = 18, 21 %) and four authors (*n* = 16, 19 %) with an arithmetic mean of 3.45. See Table 4.

**Table 4:** Authors (number and country of origin and affiliation) of the reviews

Authors				
Number of authors	Reviews (*n* = 84)	Number of authors (cont.)		Reviews (*n* = 84)
2	26 (31 %)	8		4 (5 %)
3	18 (21 %)	10		2 (2 %)
4	16 (19 %)	6		2 (2 %)
5	7 (9 %)	9		1 (1 %)
1	7 (9 %)	7		1 (1 %)
Country of origin	Reviews (*n* = 84)	Country of origin (cont.)		Reviews (*n* = 84)
USA	20 (24 %)	Croatia		1 (1 %)
Belgium	10 (12 %)	Ethiopia		1 (1 %)
United Kingdom	10 (12 %)	France		1 (1 %)
Germany	8 (10 %)	Italy		1 (1 %)
The Netherlands	6 (8 %)	Kenya		1 (1 %)
Australia	5 (6 %)	Lebanon		1 (1 %)
Canada	3 (4 %)	Mexico		1 (1 %)
Finland	3 (4 %)	South Africa		1 (1 %)
Iran	3 (4 %)	Spain		1 (1 %)
Greece	2 (2 %)	Switzerland		1 (1 %)
Ireland	2 (2 %)	Turkey		1 (1 %)
Brazil	1 (1 %)			
Affiliation (sorted after highest total)	First author (*n* = 84)	Last author (*n* = 77)	Other authors (*n* = 44)	Total (*n* = 205)
Bioethics	29 (35 %)	28 (36 %)	3 (7 %)	60 (29 %)
Medicine	18 (22 %)	16 (21 %)	17 (39 %)	51 (26 %)
Nursing/AHP	11 (13 %)	7 (9 %)	5 (11 %)	23 (11 %)
Health Science	7 (8 %)	6 (8 %)	5 (11 %)	18 (9 %)
Public Health/Global Health/International Health	4 (5 %)	3 (4 %)	2 (5 %)	9 (4 %)
Philosophy/Humanities	2 (2 %)	3 (4 %)	2 (5 %)	7 (3 %)
Genetics	1 (1 %)	0 (0.0 %)	4 (9 %)	5 (2 %)
IT/Communication	2 (2 %)	1 (1 %)	1 (2 %)	4 (2 %)
Law/Politics	1 (1 %)	2 (3 %)	1 (2 %)	4 (2 %)
Statistics	0 (0.0 %)	1 (1 %)	1 (2 %)	2 (1 %)
Other*	9 (11 %)	8 (10 %)	3 (7 %)	20 (10 %)
Not stated	0 (0.0 %)	2 (3 %)	0 (0.0 %)	2 (1 %)

*Other affiliations: Social Science, Health Education and Promotion, Institute for Quality and Efficiency in Healthcare (*IQWiG*), Populations Services Int., Corporations, Nutrition, Laboratories, Risk Management, World Health Organizations, or not specified

Twenty reviews (24 %) were written by authors from the USA, 10 (12 %) from the UK, 10 (12 %) from Belgium, 8 (10 %) from Germany, and 6 (8 %) from the Netherlands. The remaining 30 reviews were written by authors from 18 other countries. See Table 4.

We analyzed the affiliation of 205 authors with different affiliations. The greatest number, namely 60 (30 %), were affiliated to Bioethics institutions, 51 (25 %) to institutions related to medicine, 23 (11 %) to Nursing and Allied Health Practitioners (AHP)-related institutions, 18 (9 %) to Health Sciences institutions, and 7 (3 %) were affiliated to Philosophy and the Humanities. See Table 4.

### Standards/guidelines and limitations

Twenty (24 %) of the 84 reviews stated that they used an established/published review methodology (see Table 5). Only the approach of McCullough et al. and Garrar﻿d﻿ were mentioned more than once (*n* = 9, 45 %, *﻿n﻿ *= ﻿2, 10 ﻿%). Ten reviews (12 %) stated that they took guidance from established reporting standards or guidelines (whether general or specific to SRs). The only standard mentioned more than once was PRISMA, with 8 entries. Thirty-three reviews (39 %) reported on limitations.

**Table 5:** Review methodology (if explicitly stated) of the reviews

Review methodology stated (*n = 20)*		
Guideline or handbook	Year	Quotes
Higgins JPT, Green S (editors) [23]	2011	1 (5 %)
Stroup DF et al. [24]	2000	1 (5 %)
Institute of Medicine [25]	2011	1 (5 %)
Published methodological approaches		
McCullough LB et al. [9]	2007	9 (45 %)
Garrard J [26]	1999	2 (10 %)
Aveyard H [27]	2012	1 (5 %)
Jesson J et al. [28]	2011	1 (5 %)
Cen﻿tre for Reviews and Dissemination [29]	2008	1 (5 %)
Hofmann B [30]	2005	1 (5 %)
Strech D et al. [17]	2012	1 (5 %)
Whittemore R et al. [31]	2005	1 (5 %)

### Reported methods for search, selection, analysis, and synthesis

Table 6 presents detailed data on how often the reviews were transparent about methodological criteria for search, selection, analysis, and synthesis. Table 6 also highlights how these criteria match with reporting items mentioned in PRISMA. Most reviews reported, for example, on what databases (93 %), search terms (91 %), or inclusion/exclusion criteria (81 %) they used. Overall, only 1 % and 8 % did not fulfill any criteria related to search and selection, respectively. However, only a minority reported on other essential details such as the procedure for information extraction (37 %) and information synthesis (18 %). In fact, 31 % did not fulfill any criteria related to the reporting of analysis methods. For example, only 25 % of the reviews reported the ethical approach needed to analyze and synthesize normative information.

**Table 6:** Methodological criteria fulfillment of the reviews (*n* = 84)

Search method		Selection method		Analysis method		Synthesis method	
	*n* =		*n* =		*n* =		*n* =
Statement of used databases (PRISMA item 7)	78 (93 %)	Statement of inclusion and/or exclusion criteria (PRISMA item 6)	68 (81 %)	Statement of identification of information unit (e.g., definition of what information is to be extracted) (PRISMA item 10)	55 (66 %)	Statement/description of a synthesis method (PRISMA item 14)	48 (57 %)
Statement of date/period of the search(es) (PRISMA item 7)	23 (27 %)						
		Statement of the selection procedure (e.g., who was selected and how) (PRISMA item 9)	29 (35 %)				
						Statement of procedure of (employing the) synthesis method (e.g., one or two people, dialogical processes) (PRISMA item 14)	15 (18 %)
Statement of used search terms and/or search strings (PRISMA item 8)	76 (91 %)			Statement of the procedure of information extraction (PRISMA item 10)	31 (37 %)		
		Representation of search procedure as a flowchart (PRISMA item 17)	24 (29 %)				
Statement of used search restrictions (e.g., publication dates, languages) (PRISMA item 8)	50 (59 %)						
						Statement of found/included study/paper characteristics (PRISMA item 18)	45 (54 %)
				Statement of the kind of theoretical (ethical) approaches used for defining information units (PRISMA item 11)	21 (25 %)		
How many hits found (PRISMA item 17)	50 (59 %)	How many hits included (PRISMA item 17)	63 (75 %)				
						Illustration/representation of a synthesis result (PRISMA item 21)	63 (75 %)
Statement of additional search strategies used (additional criteria)	50 (59 %)						
*No criteria fulfilled*	1 (1 %)	*No criteria fulfilled*	7 (8 %)	*No criteria fulfilled*	26 (31 %)	*No criteria fulfilled*	8 (10 %)
*All criteria fulfilled*	7 (8 %)	*All criteria fulfilled*	18 (21 %)	*All criteria fulfilled*	7 (8 %)	*All criteria fulfilled*	9 (11 %)

A comprehensive qualitative analysis and comparison of all applied methods for search, selection, analysis, and synthesis is beyond the scope of this paper and is to be published elsewhere. The applied methods for search and selection of relevant normative literature are largely comparable with standard “systematic review” methodology. Methods for analysis and synthesis of normative information, however, are of substantial differences. In the following, therefore, we highlight some core findings with regard to the reported analysis and synthesis.

Regarding extraction and analysis of normative information, the most sought types of information were ethical issues, topics, or dilemmas (*n* = 27), arguments or reasons (*n* = 14), and ethical principles, values, or norms (*n* = 13) (multiple responses possible). Among the procedures for extracting information we broadly distinguished between “coding and categorizing” (*n* = 9), “collecting” (*n* = 7), or “close reading” (*n* = 6). See Table 7 for more detailed explanations and case examples.

**Table 7:** Methodological elements of analyzing normative ethical information

Information units	Explanation	Example
Ethical issues/topics/dilemmas	Overarching category for actions or situations where something has to be considered because of ethical reasons (or principles and values), or is an object of ethical research (e.g., justice in regard to disabled persons; data protection when using ambient assisted living technology; risk-benefit assessment in clinical trials; dilemmas in triage situations)	1. “The objective of the present work was to identify studies (documents, books, journals, or individual articles) that deal with disability with reference to justice and rights, in the light of the ICF. An attempt was also made to assess in statistical terms the presence of these topics in research on disability” [32] 2. “Studies were completely searched for, read and assessed according to the script made of data with the characteristics of the study and to the ethical issues raised. Ethical aspects approached were raised in the selected articles and the empirical characteristics were interpreted and organized. Analysis was performed comparing the ethical dilemmas found and that reflected upon nursing practice, based on the literature.”[33]
Ethical arguments/reasons	Normative justifications or refutations for moral claims or action plans (e.g., reasons why post-trial access should be endorsed; arguments for not telling a patient of incidental findings; arguments for allowing sexual contacts of institutionalized elderly persons).	1. “(1) What are the primary positions addressing conscientious objection to act on end-of-life procedures? and (2) upon what arguments are these positions based?” / “We analyzed the twenty-eight articles using our guiding questions and searched out common patterns in position and argumentation.” [34] 2. “To survey the main objections to the RCT and its alternatives.” [35]
Ethical principles/values/norms	Normative and theoretical concepts that summarize or describe specific ideas about ethical behavior or define a prerequisite for ethical judgement (e.g., the concept of “informed consent,” the principle of respecting patient autonomy, the ban on reproductive cloning)	1. “[…] to assemble a reliable and comprehensive account of the facts of the matter and to identify and clarify concepts that are relevant to the valuation of the ethical implications of those facts’ […]” [36] 2. “Our review identified nine different ethical frameworks outlining circumstances in which a health professional is justified in overriding parents’ medical decision-making for children. Each framework was centred on a different moral concept, such as harm or best interests. […] [12]
Ethical approach	Explanation	Example
Principlism	Widely accepted ethical approach put forward especially by T. Beauchamp and J. Childress that defines four mid-level principles that are *prima facie* binding: respect for autonomy, beneficence, non-maleficence, and justice	1. “Every source identified through the database queries was assessed for the presence of material related to each of the 4 specific ethical principles […].” [37] 2. “We collected data for each of the four ethical principles and reported major and minor themes separately […]” [38]
Pluralistic approach	Any approach that does not utilize one specific theory, but uses various theories/ approaches that can consist of principles/norms/values, etc.	“In order to capture the empirically grounded aspects of health care on which DRGs are likely to have an influence, as well as to present these aspects in light of a normative framework of ethical values, we systematically analysed the results from research on DRGs (step 1) in light of the review of ethical frameworks (step 2), and vice versa. […]”. [39]
Procedure for information extraction	Explanation	Example
Coding and categorizing	Qualitative method where information is marked under a specific “heading” (coding) and is later subsumed under more broader topics (categorizing)	1. “We made notes on each publication related to the descriptive categories and assigned each a qualitative code. […] The publications were then categorized according to decade […], ethical approach […], component of morality, topic or ethical principle […], and primary role of the audiologist as described in the literature […]” [40] 2. “From every included document we retrieved the quotes that contained recommendations or opinions on living kidney donation by minors and each quote was assigned one or more codes.” [41]
Collecting	Descriptively adding instances of the sought information without (substantial) coding and without categorizing (though possibly sorting)	1. “Articles available were screened for definitions […], definitions were extracted using the extraction tabloid […]. This resulted in a list of ends and means constitutive for PM.” [42] 2. “S.N. collected the provided arguments in the papers and recorded them in an argumentative scheme.” [43]
Close reading	Hermeneutical method of analyzing a text by examining in detail structure, wording, argumentation, style, etc., and repeating this multiple times	1. “All the articles included were read carefully and analysed for ethical reflection and discussion regarding AT use in the home environment. […]” [44] 2. “Papers were read, then reread taking notations about authors’ decisions about treatment of participants; whether or not, according to the authors, participants should be protected, based on ethical principles or established professional guidelines; the historical context of changes in Internet technology and research practices; and the authors’ professional disciplines.” [45]

Regarding synthesis, we could broadly distinguish between qualitative methods (*n* = 44), quantitative methods (*n* = 5), and narrative/hermeneutical methods (*n* = 3). In most cases, qualitative analyses aimed to develop overarching normative issues, reasons, or principles that allowed summarizing the more detailed normative information. To do this, a variety of deductively and inductively developed category systems with main and subcategories were employed. Quantitative analyses aimed, for example, to quantify the distribution of qualitatively assessed topics. See Table 8 for more detailed explanations and case examples.

**Table 8:** Methodological elements of synthesizing normative ethical information

Method used	Explanation	Example
Qualitative analysis	In general, the qualitative methods used follow the principles of “coding and categorization” as described for the *analysis* methods in Table 7. First, normative information is marked under a specific/exclusive “heading” (*coding*) mainly reflecting the *analysis* part. Second, these codes are subsumed under more broader/inclusive “headings” (*categorizing*) mainly reflecting the *synthesis* part	1. “In the stage of data combination, all textual obtained data from the selected articles were classified and completely described. After textual data had been extracted and studied critically, the traits were separated and finalized based on the obtained definition for each value or ethical concept and finally a unique definition was obtained.” [46] 2. “Beginning at the level of abstract (when present) and proceeding to the level of full text, we divided the ethical arguments into three categories: arguments in favor of disclosure; arguments opposed to disclosure; and arguments that caution about disclosure […] ” [47]
Quantitative analysis	Use of mathematical/statistical methods for displaying, e.g., a sampling distribution of topics or a correlation analysis of topics	1. “An attempt was also made to assess in statistical terms the presence of these topics in research on disability […]“ / ”The statistical summary based on the sample selected shows […]” [32] 2. “From the final cohort of citations that met the rheumatologic and ethical criteria, the proportion addressing each Beauchamp and Childress ethical principle was reported separately. These 4 proportions were analyzed using Cochran’s Q statistic to determine if the rheumatologic/ethical literature favored the treatment of certain ethical principles. […]” [37]
Narrative or hermeneutical methods	Using methods from traditional humanities that are characterized by searching and defining meaning, reaching deep understanding and merging different perspectives, e.g., by relying on close reading, comparing different texts, taking historical contexts into account, etc.	1. “Based on a systematic literature review, a hermeneutical analysis focusing the main issues of ethics in child and adolescent psychopharmacology is provided.” [48] 2. “The information contained within relevant articles was collated in the form of a narrative review.” [49]

Thirty-eight (45 %) of the included reviews (*n* = 84) reported on at least some aspects of all four domains of the methodology (search, selection, analysis, and synthesis).

## Discussion

Most reviews reported on the essential elements for search and selection methods (e.g., databases, search terms, inclusion/exclusion) except for flowcharts (reported by only 29 %). However, reporting was much less explicit for analysis and synthesis methods. Almost one third of all reviews did not report on any essential element of the analysis methods (what information to extract and how). For example, only 25 % of reviews on normative literature reported on the kind of ethical approach/theory needed to identify relevant normative information. Only 45 % of reviews reported on all methods and could therefore be labeled as (full) systematic reviews, implying that most reviews we found are rather semi-systematic. Somehow in line with the aforementioned neglect of important method reporting is the fact that only 39 % of reviews discussed their limitations.

A limitation of our review is that we only searched the databases *PubMed, PhilPapers,* and *Google Scholar*. We restricted our search to these three databases mainly because of experiences from former systematic reviews of normative information demonstrating that most of the literature can be found in *PubMed* and *Google Scholar*, and that searching other ethics-specific databases did not add a substantial proportion of references [17]. In our review, 86 % of all included reviews were found by *PubMed* searches alone. Furthermore, all languages other than English, German, or French were excluded, but this only resulted in the exclusion of three reviews.

Our results demonstrate that most elements of searching and selecting normative literature reflect the widely accepted PRISMA recommendations. However, appropriate elements for the analysis and synthesis of normative literature are less standardized. Further meta-research and conceptual analysis are needed to inform the development of minimal standards for the analysis and synthesis of normative literature. The quality assessment of normative literature might be one of the most controversial topics in this regard [10]. The required degree of transparency for all steps of information processing in analyzing and synthesizing normative information will be another controversial topic, because strong requirements in this regard might result in excessive workloads for review authors [18].

Nevertheless, our review demonstrates that analysis and synthesis methods can be described and justified with regard to the specific review objectives. This demands that the following elements for analysis and synthesis should be clarified prior to each review of normative information and should be reported with the dissemination of results: (1) normative information unit (e.g., ethical issues, ethical reasons, ethical norms, etc.), (2) ethical approach (e.g., a specific ethical theory) and the technical procedure used to identify and extract the relevant normative information units, (3) method for synthesizing normative information (e.g., category building). See Tables 7 and 8. Researchers should also be aware that these three steps are interrelated; i.e., that using a specific ethical approach will lead to a specific way of identifying normative information units, or, vice versa, that the set of normative information units identified will depend on the ethical approach (e.g., a deontological ethical theory would identify some issues as “ethical issues,” which a consequentialist ethical theory would not).

Thus, future clarification is also needed for the personal competencies and skills necessary to realize a valid and informative review of normative information. Based on our personal experiences with reviews of normative information, it is also important to clarify the expectations and needs of the intended readership. In particular, the choice of synthesis methods for normative information might differ substantially if the review group aims to inform either expert discourse in bioethics or policy decision making in guideline or HTA development. Stakeholder orientation, therefore, is another issue that should be clarified prior to conducting ethics reviews.

## Conclusions

This is the first study, to our knowledge, to analyze the state of systematic and semi-systematic reviews of normative literature on medical topics. We identified 84 reviews published between 1997 and 2015 in 65 different journals and demonstrated an increasing publication rate for this type of review. The reference lists for all included reviews (Additional file 1: Tables S1–S3) provide a rich source for those interested in medical ethics and those wanting to conduct (systematic) reviews of normative literature themselves.

Further research as well as interdisciplinary discussion and consent are needed to define detailed best practice recommendations for the respective steps of a review of normative information. Experts from different fields such as bioethics, HTA and guideline development, as well as health care professionals and patient representatives, should work together to further develop the methodology of (systematic) reviews of normative ethical information to support evidence-based health care.

## Additional file

Additional file 1: Table S1. Reviews (English/German/French): empirical literature. **Table S2**: Reviews (English/German/French): normative literature. **Table S3**: Reviews (English/German/French): mixed literature. (DOCX 56 kb)
