# Supplementary material for: Current state of ethics literature synthesis: a systematic review of reviews
Source: BMC Med. 2016 Oct 3;14:152. doi: 10.1186/s12916-016-0688-1 (PMC5052713; doi:10.1186/s12916-016-0688-1)
Supplement: Additional file 1: Table S1. — Reviews (English/German/French): empirical literature. Table S2: Reviews (English/German/French): normative literature. Table S3: Reviews (English/German/French): mixed literature. (DOCX 56 kb) [file 12916_2016_688_MOESM1_ESM.docx]

Additional file

Table S1: Reviews (English/German/French): **empirical** literature

| ***Year*** | ***Title*** | ***Authors*** | ***Journal/Link*** |
| --- | --- | --- | --- |
| 2015 | Assisted dying in dementia: a systematic review of the international literature on the attitudes of health professionals, patients, carers and the public, and the factors associated with these | Tomlinson E  Stott J | *International Journal of Geriatric Psychiatry* 30(1):10-20  www.ncbi.nlm.nih.gov/pubmed/25043718 |
| 2015 | Open-Identity Sperm Donation: How Does Offering Donor-Identifying Information Relate to Donor-Conceived Offspring's Wishes and Needs? | Ravelingien A  Provoost V  Pennings G | *Journal of Bioethical Inquiry* [Epub ahead of print]  www.ncbi.nlm.nih.gov/pubmed/24996630 |
| 2015 | Potential consequences of clinical application of artificial gametes: a systematic review of stakeholder views | Hendriks S  Dondorp W  de Wert G  et al | *Human Reproduction Update* pii: dmv002  www.ncbi.nlm.nih.gov/pubmed/25609402 |
| 2015 | Scientists Admitting to Plagiarism: A Meta-analysis of Surveys | Pupovac V  Fanelli D | *Science and Engineering Ethics* [Epub ahead of print]  www.ncbi.nlm.nih.gov/pubmed/25352123 |
| 2015 | The expectations and attitudes of patients with chronic kidney disease toward living kidney donor transplantation: a thematic synthesis of qualitative studies | Hanson CS  Chadban SJ  Chapman JR  et al | *Transplantation* 99(3):540-554  www.ncbi.nlm.nih.gov/pubmed/25463967 |
| 2014 | A mixed-method systematic review: support for ethical competence of nurses | Poikkeus T  Numminen O  Suhonen R  et al | *Journal of Advanced Nursing* 70(2):256-271  www.ncbi.nlm.nih.gov/pubmed/23865484 |
| 2014 | Can quality from a care ethical perspective be assessed? A review | Kuis EE  Hesselink G  Goossensen A | *Nursing Ethics* 21(7):774-793  www.ncbi.nlm.nih.gov/pubmed/24106258 |
| 2014 | Current practice of public involvement activities in biomedical research and innovation: a systematic qualitative review | Lander J  Hainz T  Hirschberg I  et al | *PloS One* 9(12):e113274  www.ncbi.nlm.nih.gov/pubmed/25469705 |
| 2014 | Does argumentation matter? A systematic literature review on the role of argumentation in doctor-patient communication | Labrie N  Schulz PJ | *Health Communication* 29(10):996-1008  www.ncbi.nlm.nih.gov/pubmed/24359318 |
| 2014 | Ethics and Community Involvement in Syntheses Concerning American Indian, Alaska Native, or Native Hawaiian Health: A Systematic Review | Gribble MO  Around Him DM | *AJOB Empirical Bioethics* 5(2):1-24  www.ncbi.nlm.nih.gov/pubmed/25089283 |
| 2014 | Evaluating clinical ethics support in mental healthcare: A systematic literature review | Hem MH  Pedersen R  Norvoll R  et al | *Nursing Ethics* pii: 0969733014539783  www.ncbi.nlm.nih.gov/pubmed/25091004 |
| 2014 | Factors associated with the donation and non-donation of embryos for research: a systematic review | Samorinha C  Pereira M  Machado H  et al | *Human Reproduction Update* 20(5):641-655  www.ncbi.nlm.nih.gov/pubmed/24907125 |
| 2014 | Health inequalities and regional specific scarcity in primary care physicians: ethical issues and criteria | Stapleton G  Schröder-Bäck P  Brand H  et al | *International Journal of Public Health* 59(3):449-455  www.ncbi.nlm.nih.gov/pubmed/23880912 |
| 2014 | Motivations of physicians and nurses to practice voluntary euthanasia: a systematic review | Vézina-Im LA  Lavoie M  Krol P  et al | *BMC Palliative Care* 13(1):20  www.ncbi.nlm.nih.gov/pubmed/24716567 |
| 2014 | Nurses' experiences of ethical preparedness for public health emergencies and healthcare disasters: a systematic review of qualitative evidence | Johnstone MJ  Turale S | *Nursing & Health Sciences* 16(1):67-77  www.ncbi.nlm.nih.gov/pubmed/24635901 |
| 2014 | Teaching ethics and professionalism in plastic surgery: a systematic review | de Blacam C  Vercler CJ | *Annals of Plastic Surgery* 72(4):484-488  www.ncbi.nlm.nih.gov/pubmed/24618742 |
| 2014 | The ethical dimension of nursing care rationing: A thematic synthesis of qualitative studies | Vryonides S  Papastavrou E  Charalambous A  et al | *Nursing Ethics* pii: 0969733014551377  www.ncbi.nlm.nih.gov/pubmed/25367000 |
| 2014 | Theoretical frameworks used to discuss ethical issues in private physiotherapy practice and proposal of a new ethical tool | Drolet MJ  Hudon A | *Medicine, Health Care and Philosophy* 18(1):51-62  www.ncbi.nlm.nih.gov/pubmed/24942342 |
| 2013 | Academic dishonesty today, unethical practices tomorrow? | LaDuke RD | *Journal of Professional Nursing* 29(6):402-406  www.ncbi.nlm.nih.gov/pubmed/24267935 |
| 2013 | Ethical challenges with welfare technology: a review of the literature | Hofmann B | *Science and Engineering Ethics* 19(2):389-406  www.ncbi.nlm.nih.gov/pubmed/22218998 |
| 2013 | Factors affecting the clinical use of non-invasive prenatal testing: a mixed methods systematic review | Skirton H  Patch C | *Prenatal Diagnosis* 33(6):532-541  www.ncbi.nlm.nih.gov/pubmed/23828950 |
| 2013 | Improving understanding in the research informed consent process: a systematic review of 54 interventions tested in randomized control trials | Nishimura A  Carey J  Erwin P  et al | *BMC Medical Ethics* 14(1):28  www.ncbi.nlm.nih.gov/pubmed/23879694 |
| 2013 | Interventions for shared decision-making about life support in the intensive care unit: a systematic review | Kryworuchko J  Hill E  Murray MA  et al | *Worldviews on Evidence-Based Nursing* 10(1):3-16  www.ncbi.nlm.nih.gov/pubmed/22490044 |
| 2013 | Perceived risks around choice and decision making at end-of-life: a literature review | Wilson F  Gott M  Ingleton C | *Palliative Medicine* 27(1):38-53  www.ncbi.nlm.nih.gov/pubmed/21993804 |
| 2013 | Physician attitudes toward advanced directives: a literature review of variables impacting on physicians attitude toward advance directives | Coleman AM | *American Journal of Hospice & Palliative Care* 30(7):696-706  www.ncbi.nlm.nih.gov/pubmed/23125398 |
| 2013 | Public attitudes to death and dying in the UK: a review of published literature | Cox K  Bird L  Arthur A  et al | *BMJ Supportive & Palliative Care* 3(1):37-45  www.ncbi.nlm.nih.gov/pubmed/24644327 |
| 2013 | Systematic review of attitudes toward donation after cardiac death among healthcare providers and the general public | Bastami S  Matthes O  Krones T  et al | *Critical Care Medicine* 41(3):897-905  www.ncbi.nlm.nih.gov/pubmed/23328261 |
| 2013 | Waiver of informed consent in pediatric resuscitation research: a systematic review | Eltorki M  Uleryk E  Freedman SB | *Academic Emergency Medicine* 20(8):822-834  www.ncbi.nlm.nih.gov/pubmed/24033626 |
| 2012 | From efficacy to equity: Literature review of decision criteria for resource allocation and healthcare decisionmaking | Guindo LA  Wagner M  Baltussen R  et al | *Cost Effectiveness and Ressource Allocation* 10(1):9  www.ncbi.nlm.nih.gov/pubmed/22808944 |
| 2012 | Informed consent for record linkage: a systematic review | da Silva MEM  Coeli CM  Ventura M  et al | *Journal of Medical Ethics* 38(10):639-642  www.ncbi.nlm.nih.gov/pubmed/22403083 |
| 2012 | Publishing ethics in paediatric research: A cross-cultural comparative review | Brännström I | *Nursing Ethics* 19(2):268-278  www.ncbi.nlm.nih.gov/pubmed/22457386 |
| 2012 | Nurses' decision-making in cases of physical restraint: a synthesis of qualitative evidence | Goethals S  Dierckx de Casterlé B  Gastmans C | *Journal of Advanced Nursing* 68(6):1198-210  www.ncbi.nlm.nih.gov/pubmed/22211472 |
| 2012 | What potential research  participants want to know about research: A systematic review | Kirkby HM  Calvert M  Draper H  et al | *BMJ Open* 2(3).pii: e000509  www.ncbi.nlm.nih.gov/pubmed/22649171 |
| 2012 | Whose consent matters? Controlled donation after cardiac death and premortem organ-preserving measures | Bastami S  Krones T  Biller-Andorno N | *Transplantation* 93(10):965-969  www.ncbi.nlm.nih.gov/pubmed/22576161 |
| 2011 | A systematic review of research on the meaning, ethics and practices of authorship across scholarly disciplines | Marušić A  Bošnjak L  Jerončić A | *PLoS One* 6(9):e23477  www.ncbi.nlm.nih.gov/pubmed/21931600 |
| 2011 | A systematic review of the empirical literature evaluating IRBs: what we know and what we still need to learn | Abbott L  Grady C | *Journal of Empirical Research on Human Research Ethics* 6(1):3-19  www.ncbi.nlm.nih.gov/pubmed/21460582 |
| 2011 | Burnout in palliative care: a systematic review | Pereira SM  Fonseca AM  Carvalho AS | *Nursing Ethics* 18(3):317-326  www.ncbi.nlm.nih.gov/pubmed/21558108 |
| 2011 | Ethical decision making in the resuscitation of extremely premature infants: the health care professional's perspective | Weir M  Evans M  Coughlin K | *Journal of Obstetrics and Gynaecology Canada* 33(1):49-56  www.ncbi.nlm.nih.gov/pubmed/21272437 |
| 2011 | Ethics of human genetic studies in sub-saharan Africa: the case of Cameroon through a bibliometric analysis | Wonkam A  Kenfack MA  Muna WF  et al | *Developing World Bioethics* 11(3):120-127  http://www.ncbi.nlm.nih.gov/pubmed/21781234 |
| 2011 | Inclusion and exclusion in nutrigenetics clinical research: ethical and scientific challenges | Hurlimann T  Stenne R  Menuz V  et al | *Journal of Nutrigenetics and Nutrigenomics* 4(6):322-343  www.ncbi.nlm.nih.gov/pubmed/22301706 |
| 2011 | Organizational ethics: a literature review | Suhonen R  Stolt M  Virtanen H  et al | *Nursing Ethics* 18(3):285-303  www.ncbi.nlm.nih.gov/pubmed/21558106 |
| 2011 | Prevalence of depression in granted and refused requests for euthanasia and assisted suicide: a systematic review | Levene I  Parker M | *Journal of Medical Ethics* 37(4):205-211  www.ncbi.nlm.nih.gov/pubmed/21278132 |
| 2010 | Ethical and practical concerns of surveillance technologies in residential care for people with dementia or intellectual disabilities: an overview of the literature | Niemeijer AR  Frederiks BJ  Riphagen II  et al | *International Psychogeriatics.* 22(7):1129-1142 |
| 2010 | Ethical considerations in the collection of genetic data from critically ill patients: what do published studies reveal about potential directions for empirical ethics research? | Freeman BD  Kennedy CR  Frankel H  et al | *The Pharmacogenomics Journal* 10(2):77-85  www.ncbi.nlm.nih.gov/pubmed/19997084 |
| 2010 | Ethical dilemmas concerning decision-making within health care leadership: a systematic literature review | Zydziūnaite V  Suominen T  Astedt-Kurki P  et al | *Medicina (Kaunas)* 46(9):595-603  www.ncbi.nlm.nih.gov/pubmed/21252593 |
| 2010 | Literature review: status and trends of research ethics in Swedish nurses' dissertations | Kjellström S  Fridlund B | *Nursing Ethics* 17(3):383-392  www.ncbi.nlm.nih.gov/pubmed/20444779 |
| 2010 | Nurses' ethical reasoning and behaviour: a literature review | Goethals S  Gastmans C  Dierckx de Casterlé B | *International Journal of Nursing Studies* 47(5):635-650  www.ncbi.nlm.nih.gov/pubmed/20096413 |
| 2010 | Research on ethics in nursing care for older people: a literature review | Suhonen R  Stolt M  Launis V  et al | *Nursing Ethics* 17(3):337-352  www.ncbi.nlm.nih.gov/pubmed/20444775 |
| 2009 | Are physicians willing to ration health care? Conflicting findings in a systematic review of survey research | Strech D  Persad G  Markmann G  et al | *Health Policy* 90:113-124www.ncbi.nlm.nih.gov/pubmed/19070396 |
| 2009 | Ethics Education in Surgical Residency Programs: A Review of the Literature | Helft PR  Eckles RE  Torbeck L | *Journal of Surgical Education* 66(1):35-42  www.ncbi.nlm.nih.gov/pubmed/19215896 |
| 2009 | Improving the methodologic and ethical validity of best supportive care studies in oncology: lessons from a systematic review | Cherny NI  Abernethy AP  Strasser F  et al | *Journal of Clinical Oncology* 27(32):5476-5486  www.ncbi.nlm.nih.gov/pubmed/19564538 |
| 2009 | Is it appropriate, or ethical, to use health data collected for the purpose of direct patient care to develop computerized predictive decision support tools? | Bonney W | *Studies in Health Technology and Informatics* 143:115-121  www.ncbi.nlm.nih.gov/pubmed/19380924 |
| 2009 | Nurses’ codes of ethics in practice and education: a review of the literature | Numminen O  Van Der Arend A  Helena Leino-Kilpi H | *Scandinavian Journal of Caring Sciences* 23(2):380-394  onlinelibrary.wiley.com/doi/10.1111/j.1471-6712.2008.00608.x/full |
| 2009 | Nurses' perceptions of ethical issues in the care of older people | Rees J  King L  Schmitz K | *Nursing Ethics* 16(4):436-452  www.ncbi.nlm.nih.gov/pubmed/19528101 |
| 2008 | How physicians allocate scarce resources at the bedside: A systematic review of qualitative studies | Strech D  Synofzik M  Marckmann G | *Journal of Medicine and Philosophy* 33:80-99  www.ncbi.nlm.nih.gov/pubmed/18420552 |
| 2008 | Ethics in neonatal pain research | Axelin A  Salanterä S | *Nursing Ethics* 15(4):492-499  www.ncbi.nlm.nih.gov/pubmed/18515438 |
| 2008 | Nurses' attitudes towards artificial food or fluid administration in patients with dementia and in terminally ill patients: a review of the literature | Bryon E  Dierckx de Casterlé B  Gastmans C | *Journal of Medical Ethics* 34(6):431-436  www.ncbi.nlm.nih.gov/pubmed/18511614 |
| 2008 | Nurses' moral sensitivity and hospital ethical climate: a literature review | Schluter J  Winch S  Holzhauser K  et al | *Nursing Ethics* 15(3):304-321  www.ncbi.nlm.nih.gov/pubmed/18388166 |
| 2007 | A new prescription for empirical ethics research in pharmacy: a critical review of the literature | Cooper RJ  Bissell P  Wingfield J | *Journal of Medical Ethics* 33(2):82-86  www.ncbi.nlm.nih.gov/pubmed/17264193 |
| 2007 | Institutional ethics policies on medical end-of-life decisions: a literature review | Lemiengre J  Dierckx de Casterlé B  Van Craen K  et al | *Medical Education* 83(2-3):131-143  www.ncbi.nlm.nih.gov/pubmed/17661891 |
| 2007 | Relational ethics and advocacy in nursing: literature review | MacDonald H | *Journal of Advanced Nursing* 57(2):119-126  www.ncbi.nlm.nih.gov/pubmed/17214748 |
| 2006 | A systematic literature review of the effectiveness of non-pharmacological interventions to prevent wandering in dementia and evaluation of the ethical implications and acceptability of their use | Robinson L  Hutchings D  Corner L  et al | *Health Technology Assessment* 10(26):iii, ix-108  www.ncbi.nlm.nih.gov/pubmed/16849002 |
| 2006 | The accuracy of surrogate decision makers: a systematic review | Shalowitz DI  Garrett-Mayer E  Wendler D | *Archives of Intern Medicine* 166(5):493-497  www.ncbi.nlm.nih.gov/pubmed/16534034 |
| 2005 | A systematic review of practice standards and research ethics in technology-based home health care intervention programs for older adults | Marziali E  Serafini JM  McCleary L | *Journal of Aging and Health* 17(6):679-696  www.ncbi.nlm.nih.gov/pubmed/16377767 |
| 2005 | Attitudes of academic and clinical researchers toward financial ties in research: A systematic review | Glaser BE  Bero LA | *Science and Engineering Ethics* 11(4):553-573  www.ncbi.nlm.nih.gov/pubmed/16279755 |
| 2005 | Attitudes towards carrier testing in minors: a systematic review | Borry P  Fryns FP  Schotsmans P  et al | *Genetic Counseling* 16(4):341-352  www.ncbi.nlm.nih.gov/pubmed/16440876 |
| 2005 | Risk assessment for inherited susceptibility to cancer: a review of the psychosocial and ethical dimensions | Lee RC  Kmet L  Cook LS  et al | *Genetic Testing* 9(1):66-79  www.ncbi.nlm.nih.gov/pubmed/15857189 |
| 2005 | The complexity of nurses' attitudes toward euthanasia: a review of the literature | Berghs M  Dierckx de Casterlé B  Gastmans C | *Journal of Medical Ethics* 31(8):441-446  www.ncbi.nlm.nih.gov/pubmed/16076966 |
| 2004 | Ethical aspects in the management of the terminally ill patient in the pediatric intensive care unit | Torreão Lde A  Pereira CR  Troster E | *Revista do Hospital das Clinicas Fac Med Sao Paulo* 59(1):3-9  www.ncbi.nlm.nih.gov/pubmed/15029279 |
| 2004 | Nursing resistance as ethical action: literature review | Peter E  Lunardi VL  Macfarlane A | *Journal of Advanced Nursing* 46(4):403-416  www.ncbi.nlm.nih.gov/pubmed/15117352 |
| 2003 | Reporting of informed consent and ethics committee approval in genetics studies of stroke | Meschia JF  Merino JG | *Journal of Medical Ethics* 29(6):371-a-372  www.ncbi.nlm.nih.gov/pubmed/14662820 |
| 2001 | Methodological quality and reporting of ethical requirements in clinical trials | Ruiz-Canela M  de Irala-Estevez J  Martínez-González MA  et al | *Journal of Medical Ethics* 27(3):172-176  www.ncbi.nlm.nih.gov/pubmed/11417024 |
| 1999 | Factors related to providers' decisions for and against withholding or withdrawing nutrition and/or hydration in adult patient care | Mahoney MA  Riley JM  Fry ST  et al | *The Online Journal of Knowledge Synthesis for Nursing* 6:4  www.ncbi.nlm.nih.gov/pubmed/12870092 |
| 1998 | Getting meaningful informed consent from older adults: a structured literature review of empirical research | Sugarman J  McCrory DC  Hubal RC | *Journal of the American Geriatrics Society* 46(4):517-524  www.ncbi.nlm.nih.gov/pubmed/9560079 |
| 1998 | Informed consent for clinical trials: in search of the "best" method | Edwards S  Lilford RJ  Thornton J  et al | *Social Science & Medicine* 47(11):1825-1840  www.ncbi.nlm.nih.gov/pubmed/9877351 |
| 1997 | A systematic review of empirical research into ethics in general practice | Rogers WA | *British Journal of General Practice* 47(424):733-737  www.ncbi.nlm.nih.gov/pubmed/9519523 |

Table S2: Reviews (English/German/French): **normative** literature

| ***Year*** | ***Title*** | ***Authors*** | ***Journal/Link*** |
| --- | --- | --- | --- |
| 2015 | Ethical and methodological issues in qualitative health research involving children: A systematic review | Huang X  O'Connor M  Ke L-S  et al | *Nursing Ethics* epub ahead of time  www.ncbi.nlm.nih.gov/pubmed/25552586 |
| 2015 | Ethical issues experienced by healthcare workers in nursing homes: literature review | Preshaw DH Brazil K  McLaughlin D  Frolic A | *Nursing Ethics* pii: 0969733015576357  www.ncbi.nlm.nih.gov/pubmed/25870176 |
| 2015 | Moral distress: a review of the argument-based nursing ethics literature | McCarthy J  Gastmans C | *Nursing Ethics* 22(1):131-152  www.ncbi.nlm.nih.gov/pubmed/25505098 |
| 2015 | Pragmatic randomized trials in drug development pose new ethical questions: a systematic review | Kalkman S  van Thiel GJ  Grobbee DE  et al | *Drug Discovery Today* pii:S1359-6446(15)00113-0  www.ncbi.nlm.nih.gov/pubmed/25794600 |
| 2015 | Reconsidering the ethics of sham interventions in an era of emerging technologies | Niemansburg SL  van Delden JJ  Dhert WJ  et al | *Surgery* 157(4):801-10  www.ncbi.nlm.nih.gov/pubmed/25704427 |
| 2014 | Ethical Considerations of Community-based Participatory Research: Contextual Underpinnings for Developing Countries | Jamshidi E  Morasae EK  Shahandeh K  et al | *International Journal of Preventive Medicine* 5(10):1328-1336  www.ncbi.nlm.nih.gov/pubmed/25400893 |
| 2014 | Ethical Issues in Patient Safety Research: A Systematic Review of the Literature | Whicher DM  Kass NE  Audera-Lopez C  et al | *Journal of Patient Safety* [Epub]  www.ncbi.nlm.nih.gov/pubmed/24618642 |
| 2014 | Ethics support in institutional elderly care: a review of the literature | van der Dam S  Molewijk B  Widdershoven GA  et al | *Journal of Medical Ethics* 40(9):625-631  www.ncbi.nlm.nih.gov/pubmed/24578386 |
| 2014 | Overriding parents’ medical decisions for their children: a systematic review of normative literature | McDougall RJ  Notini L | *Journal of Medical Ethics* 40(7):448-452  www.ncbi.nlm.nih.gov/pubmed/23824967 |
| 2014 | Palliative sedation therapy: a systematic literature review and critical appraisal of available guidance on indication and decision making | Schildmann EK  Schildmann J | *Journal of Palliative Medicine* 17(5):601-611  www.ncbi.nlm.nih.gov/pubmed/24809466 |
| 2014 | Patient-reported outcome (PRO) assessment in clinical trials: a systematic review of guidance for trial protocol writers | Calvert M  Kyte D  Duffy H  et al | *PloS One* 9(10):e110216  www.ncbi.nlm.nih.gov/pubmed/25333995 |
| 2014 | Perspectives from South and East Asia on clinical and research ethics: a literature review | Pratt B  Van C  Cong Y  et al | *Journal of Empirical Research on Human Research Ethics* 9(2):52-67  www.ncbi.nlm.nih.gov/pubmed/24782072 |
| 2014 | When to stop? Decision-making when children's cancer treatment is no longer curative: a mixed-method systematic review | Valdez-Martinez E  Noyes J  Bedolla M | *BMC Pediatrics* 14:124  www.ncbi.nlm.nih.gov/pubmed/24884514 |
| 2013 | [Information and consent in dental care associated with biomedical research] [Article in French] | Le Breton A  Hervé C  Pirnay P | *Santé Publique* 25(6):803-812  www.ncbi.nlm.nih.gov/pubmed/24451426 |
| 2013 | Could minors be living kidney donors? A systematic review of guidelines, position papers and reports | Thys K  Van Assche K  Nobile H  et al | *Transplant International* 26(10):949-960  www.ncbi.nlm.nih.gov/pubmed/23560654 |
| 2013 | Ethical community-engaged research: a literature review | Mikesell L  Bromley E  Khodyakov D | *American Journal of Public Health* 103(12):e7-e14  www.ncbi.nlm.nih.gov/pubmed/24134352 |
| 2013 | Informed consent for whole-genome sequencing studies in the clinical setting. Proposed recommendations on essential content and process | Ayuso C  Millán JM  Mancheño M  et al | *European Journal of Human Genetics* 21(10):1054-1059  www.ncbi.nlm.nih.gov/pubmed/23321621 |
| 2013 | The full spectrum of ethical issues in dementia care: systematic qualitative review | Strech D  Mertz M  Knueppel H  et al | *British Journal of Psychiatry* 202:400-406  www.ncbi.nlm.nih.gov/pubmed/23732935 |
| 2013 | To tell or not to tell? A systematic review of ethical reflections on incidental findings arising in genetics contexts | Christenhusz GM  Devriendt K  Dierickx K | *European Journal of Human Genetics* 21(3):248-255  www.ncbi.nlm.nih.gov/pubmed/22739341 |
| 2012 | Disability, human rights, and the International Classification of Functioning, Disability, and Health: systematic review | Aluas M  Colombetti E  Osimani B  et al | *American Journal of Physical Medicine & Rehabilitation* 91(13 Suppl 1):S146-154  www.ncbi.nlm.nih.gov/pubmed/22193322 |
| 2012 | Ethical issues and challenges in pressure ulcer research - the research nurses' perspective | Choo J  Blundell S  McGinnis E | *Journal of Tissue Viability* 21(4):105-108  www.ncbi.nlm.nih.gov/pubmed/22975387 |
| 2012 | Key ethical issues in pediatric research: islamic perspective, Iranian experience | Mobasher M  Salari P  Larijani B | *Iranian Journal of Pediatrics* 22(4):435-444  www.ncbi.nlm.nih.gov/pubmed/23429172 |
| 2012 | Public health ethics related training for public health workforce: an emerging need in the United States | Kanekar A  Bitto A | *Iranian Journal of Public Health* 41(4):1-8  www.ncbi.nlm.nih.gov/pubmed/23113159 |
| 2012 | Sexuality in institutionalized elderly persons: A systematic review of argument-based ethics literature | Mahieu L  Gastmans C | *International Psychogeriatrics* 24:346-357  www.ncbi.nlm.nih.gov/pubmed/21861944 |
| 2012 | Trust and trustworthiness in nursing: an argument-based literature review | Dinç L  Gastmans C | *Nursing Inquiry* 19(3):223-237  www.ncbi.nlm.nih.gov/pubmed/22050646 |
| 2011 | Determining authorship in multicenter trials: a systematic review | Dulhunty JM  Boots RJ  Paratz JD  et al | *Acta Anaesthesiologica Scandinavica* 55(9):1037-1043  www.ncbi.nlm.nih.gov/pubmed/21689076 |
| 2011 | Ethical issues in autologous stem cell transplantation (ASCT) in advanced breast cancer: a systematic literature review | Droste S  Herrmann-Frank A  Scheibler F  et al | *BMC Medical Ethics* 12:6  www.ncbi.nlm.nih.gov/pubmed/21496244 |
| 2011 | Ethics of using assistive technology in the care for community-dwelling elderly people: an overview of the literature | Zwijsen SA  Niemeijer AR  Hertogh CM | *Aging & Mental Health* 15(4):419-427  www.ncbi.nlm.nih.gov/pubmed/21500008 |
| 2011 | Quality of ethical guidelines and ethical content in clinical guidelines: the example of end-of-life decision-making | Strech D  Schildmann J | *Journal of Medical Ethics* 37(7):390-396  www.ncbi.nlm.nih.gov/pubmed/21343629 |
| 2011 | Reasons why post-trial access to trial drugs should, or need not be ensured to research participants: a systematic review | Sofaer N  Strech D | *Public Health Ethics* 4:160-184 www.ncbi.nlm.nih.gov/pubmed/21754950 |
| 2010 | A Review and Taxonomy of Argument-Based Ethics Literature regarding Conscientious Objections to End-of-Life Procedures | Wernow JR  Gastmans C | *Christian Bioethics* 16(3): 274-295  cb.oxfordjournals.org/content/16/3/274.full?sid=e4907512-2ed9-4001-940e-1aa33f2a0e05 |
| 2010 | Equality as a central concept of nursing ethics: a systematic literature review | Kangasniemi M | *Scandinavian Journal of Caring Sciences* 24(4):824-832  www.ncbi.nlm.nih.gov/pubmed/20487405 |
| 2010 | Safeguarding children's rights in psychopharmacological research: ethical and legal issues | Kölch M  Ludolph AG  lener PL  et al | *Current Pharmaceutical Design* 16(22):2398-2406  www.ncbi.nlm.nih.gov/pubmed/20513231 |
| 2009 | Biological sample collections from minors for genetic research: a systematic review of guidelines and position papers | Hens K  Nys H  Cassiman JJ  et al | *European Journal of Human Genetics* 17(8):979-990  www.ncbi.nlm.nih.gov/pubmed/19223929 |
| 2009 | Emerging ethical, legal and social issues associated with stem cell research & and the current role of the moral status of the embryo | Zarzeczny A  Caulfield T | *Stem Cells Reviews* 5(2):96-101  www.ncbi.nlm.nih.gov/pubmed/19521800 |
| 2009 | Nursing and euthanasia: A review of argument-based ethics literature | Quaghebeur T  Dierckx de Casterlé B  Gastmans C | *Nursing Ethics* 16:466-486  www.ncbi.nlm.nih.gov/pubmed/19528103 |
| 2009 | Substance use disorder genetic research: investigators and participants grapple with the ethical issues | Coors ME  Raymond KM | *Psychiatric Genetics* 19(2):83-90  www.ncbi.nlm.nih.gov/pubmed/19668113 |
| 2008 | Ethics knowledge in physical therapy: a narrative review of the literature since 2000 | Carpenter C  Richardson B | *Physical Therapy Reviews* 13(5):366-374  www.maneyonline.com/doi/pdfplus/10.1179/174328808X356393 |
| 2008 | Should public health be exempt from ethical regulations? Intricacies of research versus activity | Gitau-Mburu D | *East African Journal of Public Health* 5(3):160-162  www.ncbi.nlm.nih.gov/pubmed/19374317 |
| 2008 | The ethical junior: a typology of ethical problems faced by house officers | McDougall R  Sokol DK | *Journal of the Royal Society of Medicine* 101(2):67-70  www.ncbi.nlm.nih.gov/pubmed/18299625 |
| 2007 | Constructing a systematic review for argument-based clinical ethics literature: the example of concealed medications | McCullough LB  Coverdale JH  Chervenak FA | *Journal of Medicine and Philosophy* 32(1):65-76 www.ncbi.nlm.nih.gov/pubmed/17365446 |
| 2007 | Methodological and ethical issues in conducting qualitative research with children and young people: a literature review | Kirk S | *International Journal of Nursing Studies* 44(7):1250-1260  www.ncbi.nlm.nih.gov/pubmed/17027985 |
| 2006 | Carrier testing in minors: a systematic review of guidelines and position papers | Borry P  Fryns FP  Schotsmans P  et al | *European Journal of Human Genetics* 14(2):133-138  www.ncbi.nlm.nih.gov/pubmed/16267502 |
| 2006 | Presymptomatic and predictive genetic testing in minors: a systematic review of guidelines and position papers | Borry P  Stultiens L  Nys H  et al | *Clinical Genetics* 70(5):374-381 www.ncbi.nlm.nih.gov/pubmed/17026616 |
| 2004 | Professional codes in a changing nursing context: literature review | Meulenbergs T  Verpeet E  Schotsmans P  et al | *Journal of Advanced Nursing* 46(3):331-336  www.ncbi.nlm.nih.gov/pubmed/15066114 |
| 2003 | Monitoring ethical, legal, and social issues in developing population genetic databases | Austin MA  Harding SE  McElroy CE | *Genetics in Medicine* 5(6):451-457  www.ncbi.nlm.nih.gov/pubmed/14614397 |
| 2001 | Health ethics in Pakistan: a literature review of its present state | Hyder AA  Nadeem S | *Journal of Health, Population, and Nutrition* 19(1):6-11  www.ncbi.nlm.nih.gov/pubmed/11394185 |
| 2000 | Ethics in the laboratory examination of patients | Nyrhinen T  Leino-Kilpi H | *Journal of Medical Ethics* 26(1):54-60  www.ncbi.nlm.nih.gov/pubmed/10701173 |
| 1999 | Teaching medical ethics: a review of the literature from North American medical schools with emphasis on education | Musick DW | *Medicine, Health Care and Philosophy* 2(3):239-254  www.ncbi.nlm.nih.gov/pubmed/11080991 |
| 1998 | Autonomy in the rehabilitation of stroke patients in nursing homes. A concept analysis. | Proot IM  Crebolder HF  Abu-Saad HH  et al | *Scandinavian Journal of Caring Sciences* 12(3):139-45  www.ncbi.nlm.nih.gov/pubmed/9801636 |
| 1997 | Institutional review board approval and publication of human research results | Amdur RJ  Biddle C | *JAMA* 227(11):909-914  www.ncbi.nlm.nih.gov/pubmed/9062330 |

Table S3: Reviews (English/German/French): **mixed** literature

| ***Year*** | ***Title*** | ***Authors*** | ***Journal/Link*** |
| --- | --- | --- | --- |
| 2015 | A four-part working bibliography of neuroethics: part 2 - neuroscientific studies of morality and ethics | Darragh M  Buniak L  Giordano J | *Philosophy, Ethics and Humanities in Medicine* 10(1):2  www.ncbi.nlm.nih.gov/pubmed/25890310 |
| 2014 | A Review of Contemporary Work on the Ethics of Ambient Assisted Living Technologies for People with Dementia | Novitzky P  Smeaton AF  Chen C  et al | *Science and Engineering Ethics* [epub]  www.ncbi.nlm.nih.gov/pubmed/24942810 |
| 2014 | A systematic review of ethics knowledge in audiology (1980-2010) | Naudé AM Bornman J | *American Journal of Audiology* 23(2):151-157  www.ncbi.nlm.nih.gov/pubmed/24695796 |
| 2014 | Disclosing the truth: a dilemma between instilling hope and respecting patient autonomy in everyday clinical practice | Sarafis P  Tsounis A  Malliarou M  et al | *Global Journal of Health Science* 6(2):128-137  www.ncbi.nlm.nih.gov/pubmed/24576372 |
| 2014 | Systematically evaluating the impact of diagnosis-related groups (DRGs) on health care delivery: a matrix of ethical implications | Fourie C  Biller-Andorno N  Wild V | *Health Policy* 115(2-3):157-164  www.ncbi.nlm.nih.gov/pubmed/24388050 |
| 2014 | Technology and its ethics in nursing and caring journals: An integrative literature review | Korhonen ES  Nordman T  Eriksson K | *Nursing Ethics* pii: 0969733014549881  www.ncbi.nlm.nih.gov/pubmed/25335921 |
| 2013 | Clinical uncertainties, health service challenges, and ethical complexities of HIV "test-and-treat": a systematic review | Kulkarni SP  Shah KR  Sarma KV  et al | *American Journal of Public Health* 103(6):e14-23  www.ncbi.nlm.nih.gov/pubmed/23597344 |
| 2013 | Depression and decision-making capacity for treatment or research: a systematic review | Hindmarch T  Hotopf M  Owen GS | *BMC Medical Ethics* 14:54  www.ncbi.nlm.nih.gov/pubmed/24330745 |
| 2013 | Nursing ethical values and definitions: A literature review | Shahriari M  Mohammadi E  Abbaszadeh A  et al | *Iranian Journal of Nursing and Midwifery Research* 18(1):1-8  www.ncbi.nlm.nih.gov/pubmed/23983720 |
| 2013 | What is personalized medicine: sharpening a vague term based on a systematic literature review | Schleidgen S  Klingler C  Bertram T  et al | *BMC Medical Ethics* 14(1):55  www.ncbi.nlm.nih.gov/pubmed/24359531 |
| 2012 | National decision-making on adopting new vaccines: a systematic review | Burchett HE  Mounier-Jack S  Griffiths UK  et al | *Health Policy and Planning* 27 Suppl 2:ii62-76 |
| 2012 | Use of next generation sequencing technologies in research and beyond: are participants with mental health disorders fully protected? | Groisman IJ  Mathieu G  Godard B | *BMC Medical Ethics* 13:36  www.ncbi.nlm.nih.gov/pubmed/23256847 |
| 2012 | When physicians intervene in their relatives' health care | Scarff JR  Lippmann S | *Health Ethics Committee Forum* 24(2):127-137  www.ncbi.nlm.nih.gov/pubmed/22262264 |
| 2011 | Ethical aspects of human biobanks: a systematic review | Budimir D  Polasek O  Marusić A  et al | *Croatian Medical Journal* 52(3):262-279  www.ncbi.nlm.nih.gov/pubmed/21674823 |
| 2011 | Ethical considerations in the study of online illness narratives: a qualitative review | Heilferty CM | *Journal of Advanced Nursing* 67(5):945-953  www.ncbi.nlm.nih.gov/pubmed/21231955 |
| 2011 | Partizipative Entscheidungsfindung (PEF): eine systematische Übersichtsarbeit zu Begriffsverwendung und Konzeptionen  [Shared Decision Making (SDM): A Systematic Survey of Terminology Use and Concepts] [Article in German] | Rockenbauch K  Schildmann J | *Das Gesundheitswesen* 73(7):399-408  www.ncbi.nlm.nih.gov/pubmed/20859849 |
| 2010 | Ethical and professional challenges in mental health care in low- and middle-income countries | Hanlon C  Tesfaye M  Wondimagegn D  et al | *International Review of Psychiatry* 22(3):245-251  www.ncbi.nlm.nih.gov/pubmed/20528653 |
| 2010 | Global report on preterm birth and stillbirth (6 of 7): ethical considerations | Kelley M  Rubens CE  GAPPS Review Group | *BMC Pregnancy and Childbirth* 10 Suppl 1:S6  www.ncbi.nlm.nih.gov/pubmed/20233387 |
| 2010 | The relationship between organizational justice and workplace aggression | St-Pierre I  Holmes D | *Journal of Advanced Nursing* 66(5):1169-1182  www.ncbi.nlm.nih.gov/pubmed/20337799 |
| 2009 | A systematic review of ethical principles in the plastic surgery literature | Chung KC  Pushman AG  Bellfi LT | *Plastic and Reconstructive Surgery* 124(5):1711-1718  www.ncbi.nlm.nih.gov/pubmed/20009860 |
| 2009 | Ethical and practical challenges in implementing informed consent in HIV/AIDS clinical trials in developing or resource-limited countries | Mystakidou K  Panagiotou I  Katsaragakis S  et al | *SAHRAH J* 6(2):46-57  www.ncbi.nlm.nih.gov/pubmed/19936406 |
| 2009 | Genetic research on stored tissue samples from minors: a systematic review of the ethical literature | Hens K  Nys H  Cassiman JJ  et al | *American Journal of Medical Genetics Part A.* 149A(10):2346-2358  www.ncbi.nlm.nih.gov/pubmed/19764035 |
| 2008 | Ethical dilemmas experienced by nurses presented in nursing publications | Monteiro MA  Barbosa RC  Barroso MG  et al | *Revista latino-americana de enfermagem* 16(6):1054-1059  www.ncbi.nlm.nih.gov/pubmed/19229412 |
| 2008 | Ethics in the rheumatology literature: a systematic review | Caplan L  Hoffecker L  Prochazka AV | *Arthritis & Rheumatism* 59(6):816-821  www.ncbi.nlm.nih.gov/pubmed/18512718 |
| 2007 | The need for evidence-based research ethics: A review of the substance abuse literature | Anderson EE  DuBois JM | *Drug and Alcohol Dependence* 86(2-3):95-105  www.ncbi.nlm.nih.gov/pubmed/16930856 |
| 2005 | Best practice guidelines on informed consent for weight loss surgery patients | Sabin J  Fanelli R  Flaherty H  et al | *Obesity Research* 13(2):250-253  www.ncbi.nlm.nih.gov/pubmed/15800281 |
| 2004 | A concept analysis of dignity for older adults | Jacelon CS  Connelly TW  Brown R  et al | *Journal of Advanced Nursing* 48(1):76-83  www.ncbi.nlm.nih.gov/pubmed/15347413 |
| 2004 | Dying with dignity: the good patient versus the good death | Proulx K  Jacelon C | *The American Journal of Hospice & Palliative Care* 21(2):116-120  www.ncbi.nlm.nih.gov/pubmed/15055511 |
| 2003 | Psychopharmacological research ethics: special issues affecting US ethnic minorities | Miskimen T  Marin H  Escobar J | *Psychopharmacology* 171(1):98-104  http://www.ncbi.nlm.nih.gov/pubmed/14624328 |
| 2002 | Moral problems experienced by nurses when caring for terminally ill people: a literature review | Georges JJ  Grypdonck M | *Nursing Ethics* 9(2):155-178  www.ncbi.nlm.nih.gov/pubmed/11944206 |
| 2001 | Prenatal screening, ethics and Down's syndrome: a literature review | Alderson P | *Nursing Ethics* 8(4):360-374  www.ncbi.nlm.nih.gov/pubmed/16004090 |
| 2000 | Towards ethical guidelines for dealing with unsolicited patient emails and giving teleadvice in the absence of a pre-existing patient-physician relationship systematic review and expert survey | Eysenbach G | *Journal of Medical Internet Research* 2(1):E1  www.ncbi.nlm.nih.gov/pubmed/11720920 |
| 1997 | Implications of socio-cultural contexts for the ethics of clinical trials | Ashcroft RE  Chadwick DW  Clark SR  et al | *Health Technology Assessment* 1(9):i-iv, 1-65  www.ncbi.nlm.nih.gov/pubmed/9483158 |
